# Supplementary material for: Antenatal care booked rural residence women have home delivery during the era of COVID-19 pandemic in Gidan District, Ethiopia
Source: PLoS One. 2023 Dec 5;18(12):e0295220. doi: 10.1371/journal.pone.0295220 (PMC10697573; doi:10.1371/journal.pone.0295220)
Supplement: S1 File — (DOCX) [file pone.0295220.s001.docx]

**Amharic version questionnaires**

***መጠይቆች***

| **ክፍል-አንድ: የማህበራዊ ና የስነ-ህዝብ መጠይቅ** | | | |
| --- | --- | --- | --- |
| ተራ.ቁ | ጥያቄዎች | መልስ | ኮድ |
| 101 | የእናትየዋ እድሜ ስንት ነው? | -------- አመት |  |
| 102 | የመኖሪያ ቦታዎ የትነው ? | 1. 1= ከተማ   2=ገጠር |  |
| 103 | የትኛው ሀይማኖት ተከታይ ኖዎት? | 1=ኦርቶዶክስ  2=ሙስሊም  3=ፕሮቴስታንት  4=ካቶሊክ  5=ሌላ_____ |  |
| 104 | የእናትየዋ የት/ት ደረጃ? | 1=ያልተማረ  2=ማንበብ ና መጸፍም ትችል  3=የመጀመሪያ ደረጃ  4=ሁለተኛ ደረጃ  5=ከፍተኛ ደረጃ (ኮሌጅ እና ከዛ በላይ) |  |
| 105 | የእናትየዋ ስራ ምንድ ነው? | 1=የቤት እመቤት  2=የመንግስትሰራተኛ  3=ነጋዴ  4=የቀንሰራተኛ  5=ሌላ______ |  |
| 106 | የእናትየዋ የጋብቻ ሁኔታ? | 1=ያላገባች  2=ያገባች  3=የተፋታች  4=የሞተባት | መልስዎ ከ 2 ውጭ ከሆነ ወደ ጥያቄ ቁጥር 201 ይለፉ |
| 107 | የባልተቤትዎ የት/ት ደረጃ? | 1=ያልተማረ  2=ማንበብ ና መጸፍም ትችል  3=የመጀመሪያ ደረጃ  4=ሁለተኛ ደረጃ  5=ከፍተኛ ደረጃ (ኮሌጅ እና ከዛ በላይ) |  |
| 108 | የባልተቤትዎ ስራ? | 1= አርሶ አደር  2=የመንግስት ሰራተኛ  3=ነጋዴ  4=የቀን ሰራተኛ  5=ሌላ________ |  |
| **ክፍል ሁለት: ከማህፀንና ጤና አገልግሎቶች አጠቃቀም ጋር ተያያዥነት ያላቸው** | | | |
| **201** | በመጨረሻ እርግዝናዎ ቅድመ ወሊድ ክትትል አድርገዋል? | 1=አዎ  2=አላደረኩም |  |
| **202** | ልጅዎትን የት ነው የወለዱት? | 1-ቤት  2=ጤና ጣቢያ  3=ሆስቲታል  4=ሌላ ካለ ይጥቀሱ ____ |  |
| **203** | እርስዎ የሴቶች የልማት ሰራዊት /ሁሜን ደቨሎፕመንት አርሚይ አባል ነዎት? | 1=አዎ  2=አይደለሁም |  |
| **204** | ምን ያክል ልጅ ወለድሽ በህይወት ያሉትንም የጠፉትንም ጨምሮ | ____________በቁጥር |  |
| **205** | አሁን በህይወት ያሉት ልጆች | _________በቁጥር |  |
| **206** | በህይወት ጠፍተው የተወለደ አለዎት | 1=አወ  2=የለም |  |
| **207** | የርግዝናዎ ሁኔታ | 1=የታቀደ እርግዝና  2=ያልታቀደ ርግዝና |  |
| **208** | የናቶች የጤና አገልግሎት (ወሊድ ክትትል) ተቋርጦ ያውቃን? | 1=አወ  2=የለም |  |
| **209** | የትራንስፖርት ችግር አለ ወደ ጤና ተቁአም ሲትሄጅ | 1=አወ  2=የለም |  |
| **210** | COVID-19 እያዛለው ቢለሽ ትፈሪአለሽ ወደ ጤና ተቁአም ሲትሄጅ | 1=አወ  2=የለም |  |
