# Supplementary material for: Antenatal care booked rural residence women have home delivery during the era of COVID-19 pandemic in Gidan District, Ethiopia
Source: PLoS One. 2023 Dec 5;18(12):e0295220. doi: 10.1371/journal.pone.0295220 (PMC10697573; doi:10.1371/journal.pone.0295220)
Supplement: S2 File — (DOCX) [file pone.0295220.s002.docx]

**English version questionnaires**

Questionnaire code ________

Kebele_________

Section I: Socio-demographic characteristics of the participants

| NO. | Questions | Response | Skip |
| --- | --- | --- | --- |
| 101 | Age of the mother? | --------in year |  |
| 102 | What is your usual place of residence area | 1=rural  2=urban |  |
| 103 | What is your religion? | 1=orthodox  2=Muslim  3=protestant  4=catholic  5=other |  |
| 104 | Educational status of the mother? | 1=unable to read and write  2=able to read and write  3=primary school  4=secondary school  5=college and above  6=other(specify----) |  |
| 105 | What is your occupation? | 1= Housewife  2= Government employed  3=Private organization employed  4=Merchant  5=farmer  5= Daily laborer  6=other specify |  |
| 106 | What is your current marital status? | 1=Single  2=Married  3=Widowed  4= Divorced  5=Separated  6=other (specify….) | If not married, skip to question number 113 |
| 107 | What is your husband level of education? | 1=unable to read and write  2=able to read and write  3=primary school  4=secondary school  5=college and above  6= other(specify-----) |  |
| 108 | What is your husband occupation? | 1=farmer  2=merchant  3=government employer  4=private worker  6=other (specify-----) |  |
| **Section II: Obstetric and other related factors** | | | |
| 201 | Did you attend ANC during your last pregnancy? | 1=Yes  1=No |  |
| 202 | Where did you deliver your last child? | 1=home  2=health center  3=hospital  4=other (specify……) |  |
| 203 | Are you member of women development army(WDA) | 1=Yes  2=No |  |
| 204 | Parity | -------in number |  |
| 205 | Number of child alive | ------number |  |
| 206 | Did you ever had bad obstetric history (still birth, abortion, etc.) | 1=Yes  2=No |  |
| 207 | What was the status of last pregnancy? | 1=Planned  2= unplanned |  |
| 208 | Did you face interruption and diversion of maternal health care services during your last pregnancy | 1=Yes  2=No |  |
| 209 | Did you face lack of transport when you visit the health facilities | 1=Yes  2=No |  |
| 210 | Did you fear COVID-19 when you visit the health facilities | 1=Yes  2=No |  |
