# Supplementary material for: Antenatal care booked rural residence women have home delivery during the era of COVID-19 pandemic in Gidan District, Ethiopia
Source: PLoS One. 2023 Dec 5;18(12):e0295220. doi: 10.1371/journal.pone.0295220 (PMC10697573; doi:10.1371/journal.pone.0295220)
Supplement: Attachment — Submitted filename: point by point response.docx [file pone.0295220.s005.docx]

Authors’ response to the editor and reviewer’s comments

Title**:** Home delivery and associated factors among antenatal care booked women in their last pregnancy in Northeast Ethiopia.

Authors

Desale Bihonegn Asmamaw ([desalebihonegn1988@gmail.com](mailto:desalebihonegn1988@gmail.com))

Yohannes Ayanaw Habitu (([yohaneshabitu@gmail.com](mailto:yohaneshabitu@gmail.com))

Eskedar Getie Mekonnen ([eskedargetie18@gmail.com](mailto:eskedargetie18@gmail.com))

Wubshet Debebe Negash ([wubshetdn@gmail.com](mailto:wubshetdn@gmail.com))

Date: 08/07/2022

Authors’ Point-by-point response to editor and reviewer comments

We are very grateful to both the editor and reviewers for your comments and concerns. All the concerns raised so far will have an undeniable impact on improving the quality and readability of our scholarly work. Appreciating your effort and valuable comments, we have provided possible reflections on the raised concerns and questions. Kindly find our reflections here.

| S. no. | Comments | Authors response |
| --- | --- | --- |
|  | Reviewer 1 comments | Authors response |
| 1 | Background section-Page 4, Line 51 Of this two-thirds of the deaths from SSA I think you mean; Of this two-thirds of the deaths were from SSA | Thank you for your observations. This has been corrected. Kindly see page 4, line 51-52. |
| 2 | Page 5, line 76,77; Another study in Ethiopia revealed that many women 77 unpredictably did not deliver in health facilities despite antenatal care You should write as Another study from Ethiopia revealed that many women 77 unpredictably did not deliver in health facilities despite antenatal care | Comment accepted. This has been modified. kindly see page 5, line 76-77. |
| 3 | Study variables-Page 7, Line 121, 122 When a women gave birth at her home or others’ home 122 (neighbor, relatives, or family) or when a birth takes place outsides of health institutions. You should write, When a woman gave birth at her home or others’ home 122 (neighbor, relatives, or family) or when a birth took place outsides of health institutions | Thank you for your observation. This has been corrected. Kindly see page 7, line 121-124. |
| 4 | Result section-Page 10, Line 166, 167 306 (40.3%) of participants fall within the age category of 167 26-30 years. You should write, 306 (40.3%) of participants fell within the age category of 167 26-30 years. | Thank you for your comments and your comment is accepted. Kindly see page 10, line 175-177. |
| 5 | Page 10, Line 172, 173 About 532(70%) of the mothers were feared 173 COVID-19 infection You should write, About 532(70%) of the mothers feared 173 COVID-19 infection | Thank you for your observations. This has been addressed. Kindly see page 10, line 182-83. |
| 6 | Discussion-Pg 11, 12, Line-194, 195 a significant number of pregnant women still give birth at home, contrary to plane of health institution delivery. You should write-a significant number of pregnant women still give birth at home, contrary to plan of health institution delivery. | Comment accepted. The mistake has been corrected. Kindly see page 11, line 203-204. |
| 7 | Pg 12, Line-199, 200 This difference might be due to the effect of COVID-19 pandemic at which women had high-perceived severity. Rephrase this line | Thank you for your comments and your comment is accepted. This has been addressed accordingly. Kindly see page 12, line 210-214. |
| 8 | g 12, Line-206-209 This lower prevalence might be that time and study population difference as the current study included only those individuals who had ANC follow up which is one of the most known determinants of home delivery and the government’s ongoing effort to improve the health care system Rephrase this statement | Thank you for your suggestions. This has been rephrased. Kindly see page 12. Line 220-223 |
| 9 | Pg 12, Line-212, 213 This implied that strengthening the above activities will be resulted a better outcome. You should write-This implied that strengthening the above activities will result in a better outcome. | Thank you for your observations. This has been addressed. Kindly see page 12, line 226-227. |
| 10 | Pg 13, Line-218-220 Moreover, women form rural areas were limited decision-making autonomy, lack of knowledge of pregnancy complication and limited access to information than urban women You should write-Moreover, women from rural areas with limited decision-making autonomy, lack of knowledge of pregnancy complications, and limited access to information from urban women | Thank you for your suggestions. This has been modified accordingly. Page 13, line 232-234. |
| 11 | Pg 13, Line-229, 230 Besides, non-educated husbands do not aware the difficulty and complication happen during pregnancy and childbirth. You should write-Besides, non-educated husbands are unaware of the difficulty and complications associated with pregnancy and childbirth. | Thank you for your comments. This has been addressed. Kindly see page 13, line 243-245. |
| 12 | Pg 13, Line-234, 235 This finding 235 is in congruent with a study conducted in Southern Ethiopia [47], Write a full stop at the end. | Thank you for your observations. The grammatical error has been addressed, kindly see page 13, line 249-250. |
| 13 | Pg 13, Line-235- 237 The possible reason might be women who did not involve in WDA are less likely to discuss with health care providers and with each other about their health including where to give birth The possible reason might be that women who are not part of WDA are less likely to discuss their health issues, including place of birth among each other as well as other health care providers. |  |
| 14 | Pg 14, Line-240-246 The finding of this study revealed that fear of COVID-19 infection were another factor that affect place of delivery. Women who were feared of COVID-19 infection were 3.84 times more odds to deliver at home than women who were not feared COVID-19 infection, this finding was consistent with a study done in India. The reason behind might be low awareness about the preventive measures of COVID-19, in addition, a study conducted in Italy among pregnant women found that there were fear of visiting health facilities for delivery because of fearing COVID-19 infection You should write-The finding of this study revealed that fear of COVID-19 infection was another factor that affected the place of delivery. Women who feared COVID-19 infection had 3.84 times more odds to deliver at home than women who did not fear COVID-19 infection. This finding was consistent with a study done in India. The reason behind this might be low awareness about the preventive measures for COVID-19. In addition, a study conducted in Italy among pregnant women found that there was fear of visiting health facilities for delivery because of COVID-19 infection. | Thank you for your suggestions. This has been addressed. Kindly see page 14, line 255-260. |
| 15 | Strength and limitations-Pg 14, Line 250, 251 In addition, May introduce a 251 recall bias due to data was collected from women about their experience since 1 year back. You should write-In addition; there might be a recall bias due to data collected from women about their experiences 1 year back. | Thank you for your suggestions. We have corrected it. Kindly see page 14, line 263-265. |
| 16 | Conclusion-Pg 14, Line-258, 259 improving the husband’s educational status, providing information related to health institution delivery benefits You should write-Improving the husband’s educational status, providing information related to health institution delivery benefits | Thank you for your observations. The grammar error has been corrected. Kindly see page 14, line 272-273. |
| 17 | Page-14, 15, Line-261-264 Future researchers interested in the area better to address why pregnant women preferred to deliver in the home though qualitative approach, which might have a tremendous effect on institutional services delivery utilization. You should write-Future researchers interested in the area better address why pregnant women preferred to deliver in the home through a qualitative approach, which might have a tremendous effect on institutional service delivery utilization. | Thank you for your comments. This has been modified accordingly. Kindly see page 15, line 280-283. |
| 18 | I think you should look into table 3-home delivery variable. Were there no home deliveries as there are no numbers mentioned under the YES home-delivery variable? | Thank you for you observations. This has been addressed. Kindly see table 3. |

Thank you very much!
