# Supplementary material for: Antenatal care booked rural residence women have home delivery during the era of COVID-19 pandemic in Gidan District, Ethiopia
Source: PLoS One. 2023 Dec 5;18(12):e0295220. doi: 10.1371/journal.pone.0295220 (PMC10697573; doi:10.1371/journal.pone.0295220)
Supplement: Attachment — Submitted filename: point by point response.docx [file pone.0295220.s006.docx]

Authors’ response to the editor and reviewer’s comments

Title**:** Impact of fear of COVID-19 infection on home delivery among antenatal care booked women in Northeast Ethiopia

Authors

Desale Bihonegn Asmamaw ([desalebihonegn1988@gmail.com](mailto:desalebihonegn1988@gmail.com))

Yohannes Ayanaw Habitu (([yohaneshabitu@gmail.com](mailto:yohaneshabitu@gmail.com))

Eskedar Getie Mekonnen ([eskedargetie18@gmail.com](mailto:eskedargetie18@gmail.com))

Wubshet Debebe Negash ([wubshetdn@gmail.com](mailto:wubshetdn@gmail.com))

Date: 20/112022

Authors’ Point-by-point response to editor and reviewer comments

Dear reviewer, we would like to extend our deepest appreciation for devoting your time to reviewing our manuscript entitled “impact of fear of COVID-19 infection on home delivery among antenatal care booked women in Northeast Ethiopia ". There has been a major revision of this manuscript (Abstract, introduction, methods, results, discussion, and conclusions). The whole structure of the manuscript has been revised. We hope now the manuscript is clear and more acceptable than its previous version. We have tried to present the paper in the proper manner according to your comment on what to suppose to do so. For this, here we have given our responses to each of the concerns you raised. Again, we would like to remind our strongest gratitude for your effort in the improvement of this manuscript, and all the points were addressed in the point-by-point response.

General comments of the reviewer

Reviewer comments: Refine the title. The title should not at least briefly explain the whole content of the manuscript. You can use absorbing and attractive findings to be your title.

Authors’ response: Thank you for your suggestion, this has been modified according to the findings, kindly see the updated version of the title.

Reviewer comments: Tense flaws i.e., the background section and the result section.

Authors’ response: Thank you for your comments, this has been edited, kindly see the updated version of the manuscript.

Reviewer comments: Incorrect use of words E.g., the “…was delivered” can be written as “…had given birth”

Authors response: Thank you for your observations, this has been addressed. Kindly see the updated version of the manuscript.

Reviewer comments: Update the background with new initiatives. E.g. MOH new ANC guideline and other maternal initiatives of the ministry.

Authors’ response: Thank you for your comments, this has been addressed. Kindly see the updated version of the introduction.

Reviewer comments: You can frame the whole section of the manuscript “…. discontinuity in the continuum of maternal health care

Authors response: Dear reviewer, thank you for the comments and suggestions, this has been modified accordingly. Kindly see the updated version of the manuscript.

Reviewer comments: Tense, grammar, the content of the sections, and presentation needs further emphasis.

Authors response: Thank you for your observation and suggestions. The entire article has been read and corrected by a native English speaker. Kindly see the updated version of the manuscript.

Reviewer comments: Paragraph should not be one sentence.

Authors response: Thank you for your observations and suggestions. This has been addressed; kindly see the updated version of the manuscript.

**Specific comments**

Reviewer comments: What is the motive behind booking of ANC? Why the book first and then fail to trust the institutional delivery? Would you explain it before and after the introduction of COVID 19?

Authors response: Thank you for the comments and observations. In fact, pregnant women who had ANC visits are expected to give birth at health institutions, there are studies that found that pregnant women who had ANC visits gave birth at home (drop out from the continuum of maternal health care) [1, 2]. Home birth after ANC is a major public health issue in Ethiopia, despite the fact that few studies on the determinants of the practice have been conducted [1]. We tried to explain it before and after the introduction of COVID-19. Kindly the updated version of the introduction and limitation section.

Reviewer comments: Differentiate your literature between the ANC full care and Last booked ANC care throughout the document.

Authors response: Thank you for your suggestion, this has been differentiated. Kindly see the updated version of the document.

Reviewer comments: Where did you include in the outcome if a mother give birth on her way to health facility?

Authors response: Home delivery was defined as any birth that had taken place in the woman’s or others’ home, while deliveries that occurred in governmental health posts, health centers, hospitals, private clinics and hospitals, and NG health facilities were grouped as facility-based deliveries. If a mother gives birth on her way to health facilities, she was considered a home delivery [2].

Reviewer comments: What is known about booking and then leaving the continuum of care? What is unknown? And what do you want to fill by this study?

Authors response: Ethiopia strives to end maternal and neonatal mortalities through increased production of skilled professionals on maternal and child health, collaboration with different governmental and non-governmental organizations, increased budget allocation, and give special emphasis. In Ethiopia according to the 2016 EDHS report, 62% of women were booked for ANC and only 26% of pregnant women had given birth at health institutions. There are variety of literature reporting factors that affect the continuum of maternal health care [2, 4-10], but there is no adequate literature explaining why ANC-booked women prefer home deliveries and the impact of fear of COVID-19 infection on home delivery. This leaves maternal morbidity and mortality as a public health problem. Therefore, the aim of this study was to assess the impact of fear of COVID-19 on home delivery among women who had booked ANC. Kindly see the updated version of the introduction.

Reviewer comments: Why multi stage for a district? The sampling procedure is not clear to the reader. Is that multi stage? Is that stratified? Is that SRS?

Authors response: Multistage sampling was employed to select study subjects. First, all kebeles in the District were stratified in to urban and rural. From 23 kebeles stratified into two (21 rural kebeles and two urban kebele), six rural and one urban kebeles were selected by simple random sampling technique using the lottery method. Mothers in the sampled kebeles selected by using simple random sampling technique (open Epi Random Program version 3) [11-13]. Kindly see page 8, line 119-130, and figure 1.

Reviewer comments: You had visited three times. What is the day time you have visited the home?

Authors’ response: Dear reviewer, thank you for your comments. The data was collected from home to home; the data collectors visited the homes on working days (Monday to Friday) because, in the Ethiopian context, almost all rural women and most urban women go to church (mostly orthodox) and market on Saturday and Sunday. The data collectors returned to the home if they did not find the participants at the time. When the interviewers failed to find the eligible respondent after three visits, the next household was included.

Reviewer comments: The data quality assurance should be clearer at the three stage of the process and how it was ensured?

Authors response: Thank you for the comments and suggestions, this has been addressed in detail, kindly see page 9, line 143-152.

Reviewer comments: When did they collect the data? Is that immediately at giving birth, or after one month of their giving birth…etc?

Authors response: Thank you for your comments. Regardless of the outcome, limiting the participants to only a 1-year period and after one month of giving birth was made to minimize potential recall bias and make the mother stabilized and comfortable. Kindly see page 8, line 127-130.

Reviewer comments: Confidence interval should follow your percentage?

Authors response: thank you for your comments, this has been dressed kindly see the updated version of the manuscript.

Reviewer comments: Revisit for the contents of the sections in general

Reviewer comments: Thank you for your suggestions. We were revisited the contents of the section. Kindly see the updated version of the manuscript.

Reviewer comments: Revisits the statistics again for frequency, univariate, binary and multiple logistic regression e.g. Why P value < 0.05? P value < 0.025?

Authors response: variables at less than or equals to 0.25 *p*-values in the bivariable logistic regression model were fitted into the multivariable logistic regression model to control the effect of confounding variables. Variable having *P*-value less than or equals to 0.05 in the multivariable logistic regression analysis was considered as associated factors for factors for home delivery. The reason for using P value < 0.05? P value < 0.25? Based on reviewing literatures and there are a lot of research that are used such kinds of cut of point [2, 11, 13]. Kindly consider these kinds of issues.

Reviewer comments: Finally, you can cross tabulate women who fear infection of COVID 19 and home delivery. Why? You can even reframe the title by this

Authors response: Thank you for your comments and suggestions, this has been addressed according. Kindly see the updated version of the manuscript.

1.	Muluneh AG, Animut Y, Ayele TA: **Spatial clustering and determinants of home birth after at least one antenatal care visit in Ethiopia: Ethiopian demographic and health survey 2016 perspective**. *BMC pregnancy and childbirth* 2020, **20**(1):1-13.

2.	Kasaye HK, Endale ZM, Gudayu TW, Desta MS: **Home delivery among antenatal care booked women in their last pregnancy and associated factors: community-based cross sectional study in Debremarkos town, North West Ethiopia, January 2016**. *BMC pregnancy and childbirth* 2017, **17**(1):1-12.

3.	Berhan Y, Berhan A: **Antenatal care as a means of increasing birth in the health facility and reducing maternal mortality: a systematic review**. *Ethiopian journal of health sciences* 2014, **24**:93-104.

4.	Wodaynew T, Fekecha B, Abdisa B: **Magnitude of home delivery and associated factors among antenatal care booked mothers in Delanta District, South Wollo Zone, North East Ethiopia: a cross-sectional study, March 2018**. *Int J Womens Health Wellness* 2018, **4**(2):1-11.

5.	Tessema ZT, Tiruneh SA: **Spatio-temporal distribution and associated factors of home delivery in Ethiopia. Further multilevel and spatial analysis of Ethiopian demographic and health surveys 2005–2016**. *BMC pregnancy and childbirth* 2020, **20**(1):1-16.

6.	Kifle MM, Kesete HF, Gaim HT, Angosom GS, Araya MB: **Health facility or home delivery? Factors influencing the choice of delivery place among mothers living in rural communities of Eritrea**. *Journal of Health, Population and Nutrition* 2018, **37**(1):1-15.

7.	Delibo D, Damena M, Gobena T, Balcha B: **Status of home delivery and its associated factors among women who gave birth within the last 12 months in east Badawacho District, Hadiya zone, Southern Ethiopia**. *BioMed Research International* 2020, **2020**.

8.	Chernet AG, Dumga KT, Cherie KT: **Home delivery practices and associated factors in Ethiopia**. *Journal of reproduction & infertility* 2019, **20**(2):102.

9.	Amano A, Gebeyehu A, Birhanu Z: **Institutional delivery service utilization in Munisa Woreda, South East Ethiopia: a community based cross-sectional study**. *BMC pregnancy and childbirth* 2012, **12**(1):1-6.

10.	Ayele G, Tilahune M, Merdikyos B, Animaw W, Taye W: **Prevalence and associated factors of home delivery in Arbaminch Zuria district, southern Ethiopia: community based cross sectional study**. *Science* 2015, **3**(1):6-9.

11.	Tsegaye AT, Mengistu M, Shimeka A: **Prevalence of unintended pregnancy and associated factors among married women in west Belessa Woreda, Northwest Ethiopia, 2016**. *Reproductive health* 2018, **15**(1):1-8.

12.	Markos D, Bogale D: **Birth preparedness and complication readiness among women of child bearing age group in Goba woreda, Oromia region, Ethiopia**. *BMC pregnancy and childbirth* 2014, **14**(1):1-9.

13.	Yisak H, Gobena T, Mesfin F: **Prevalence and risk factors for under nutrition among children under five at Haramaya district, Eastern Ethiopia**. *BMC pediatrics* 2015, **15**(1):1-7.
