# Supplementary material for: Antenatal care booked rural residence women have home delivery during the era of COVID-19 pandemic in Gidan District, Ethiopia
Source: PLoS One. 2023 Dec 5;18(12):e0295220. doi: 10.1371/journal.pone.0295220 (PMC10697573; doi:10.1371/journal.pone.0295220)
Supplement: Attachment — Submitted filename: point by point response.docx [file pone.0295220.s007.docx]

Authors’ response to the reviewer’s comments

Title**:** Unmarried women have high home delivery during the era of COVID-19 in Gidan district Northeast Ethiopia

Authors

Desale Bihonegn Asmamaw ([desalebihonegn1988@gmail.com](mailto:desalebihonegn1988@gmail.com))

Yohannes Ayanaw Habitu (([yohaneshabitu@gmail.com](mailto:yohaneshabitu@gmail.com))

Eskedar Getie Mekonnen ([eskedargetie18@gmail.com](mailto:eskedargetie18@gmail.com))

Wubshet Debebe Negash ([wubshetdn@gmail.com](mailto:wubshetdn@gmail.com))

Date: 04/022023

Authors’ Point-by-point response

Dear reviewer, we would like to extend our deepest appreciation for devoting your time to reviewing our manuscript entitled “Unmarried women have high home delivery during the era of COVID-19 in Gidan district Northeast Ethiopia". There has been a revision of this manuscript (Abstract, introduction, methods, results, discussion, and conclusions). The whole structure of the manuscript has been revised. We hope now the manuscript is clear and more acceptable than its previous version. We have tried to present the paper in the proper manner according to your comment on what to suppose to do so. For this, here we have given our responses to each of the concerns you raised. Again, we would like to remind our strongest gratitude for your effort in the improvement of this manuscript, and all the points were addressed in the point-by-point response.

Reviewer comments

Reviewer comments: Revised as per comment: More than 90% have been addressed. However, still some of them needs brief inclusion into the revised paper. E.g., sentence that described “...women was had given birth” in line 39 and 40 needs meticulous edition.

Authors response: Thank you for your comments, this has been corrected accordingly. Kindly see line40-42.

Reviewer comments: We have report that states institutional service delivery was hampered as a result of fear of health institution related COVID-19 infection. Hence, what does “Impact of fear of COVID-19 infection on home delivery among antenatal care booked women” mean? Therefore, you are expected to reason out more the component of the titles and the title still needs meticulous revision ? If it is a must to continue with this type of title / I didn’t recommend/ it can be reframed as “unmarried women have high home delivery during the era of COVID-19 in Northeast Ethiopia”. The study area was wide. Hence, should be in some district or zone.

Authors response: Dear reviewer, thank you for your comment. The title was reframed as you suggested. Kindly see the updated version of the manuscript.

Reviewer comments: By the way the title and the dependent variable are inconsistent

Authors’ response: Dear reviewer, thank you for your observations. We corrected the errors. And the title and the dependent variable are consistent now. Kindly see the updated version of the manuscript.

Reviewer comments: The keywords are not complete.

Authors response: Thank you for your suggestion and observations. This has been addressed. Kindly see the updated version of the keywords.

Reviewer comments: In the background of the abstract, you have stated as “despite the importance of giving birth at health facilities, in Ethiopia, nearly half of the mothers who were booked for antenatal care gave birth at home”. Therefore, what was the evidence? Is that before or after the introduction of COVID-19? Hence, if they already half of them give birth at home before the pandemic? What is new then after the pandemics?

Authors’ response: Dear reviewer, thank you for your comments. In Ethiopia, nearly half of the mothers who were booked for antenatal care gave birth at home. This was reported by Ethiopia Demographic health survey (EDHS) of 2016. After the pandemic of COVID-19, the utilization of maternal health care like institutional delivery was more compromised [1]. This has been addressed in the updated version of the manuscript. Kindly see the updated version of the manuscript.

Reviewer comments: why they are not included in women’s development army since it is comprehensive package of care for women?

Authors’ response: Dear reviewer, thank you for your point of view. Yes, women’s development army is a comprehensive package of care for women. However, all women are not actively participated in the women’s development army. Only some of the women are actively involved in the package [2, 3]. Kindly consider these issues.

Reviewer comments: Your objective, methods, results, discussions and the conclusions are not well consistent.

Authors response: Thank you for your comments. This has been corrected accordingly. Kindly see the updated version of the manuscript.

Reviewer comments: What is the philosophical stance of the researchers?

Authors response: Thank you for your comments. Maternal morbidity and mortality are a global health challenge, and developing countries contribute to most maternal deaths. This can be reduced by improving maternal health care services utilizations like institutional service delivery. Previous scholars have stated that home delivery can be reduced by strengthening antenatal care (ANC) service utilization. But there are some studies reporting that ANC-booked women are still giving birth at home. And currently, places of delivery are also affected by the COVID-19 pandemic. Therefore, the aim of this study was to determine the prevalence and associated factors of home delivery among women who had antenatal care booked in their last pregnancy during the era of COVID-19.

Reviewer comments: Many grammatical and language errors E.g., Stata to mean “STATA”.

Authors response: Thank you for your observations. This has been corrected accordingly. Kindly see the updated version of the manuscript.

Reviewer comment: Still the data quality measures need detail emphasis?

Authors response: Thank you for your suggestions and comments. This has been addressed. Kindly see line 146-152.

Reviewer comments: What are the measures taken to reduce recall bias?

Authors response: Thank you for your comments. To reduce recall bias, some possible strategies were used, such as providing training for data collectors, employing pretests, and using standardized tools.

Reviewer comment: Did the case to variable ratio fit? How did you control confounders and multi-collinearity? Why you presented mean?

Authors response: Thank you for your comments. Prior to identifying the significant factors, multicollinearity was tested using the variance inflation factor (VIF), and we got a VIF of less than five for each independent variable with a mean VIF of 1.56, indicating there was no significant multicollinearity between independent variables. We used mean because, before selecting a summary measure, we checked for normality tests such as kurtosis and skewness were employed to see the normal distribution of the variables and to identify which summary measures were appropriate to use. We got a symmetric distribution, therefore we used mean. Kindly consider these issues.

Reviewer comment: Avoid repetitions. E.g., ‘declarations”.

Authors response: Thank you for your observations. The repetitions were avoided.

Reviewer comments: The contents, statistics and language needs English language expert before the next submission.

Authors response: Dear reviewer, thank you for your comments. An academic English language experts exhaustively proofread and edited the document. Kindly see the updated version of the manuscript.

References

1.	Abdisa DK, Jaleta DD, Feyisa JW, Kitila KM, Berhanu RD: Access to maternal health services during COVID-19 pandemic, re-examining the three delays among pregnant women in Ilubabor zone, southwest Ethiopia: A cross-sectional study. Plos one 2022, 17(5):e0268196.

2.	Wondimu MS, Woldesemayat EM: Determinants of home delivery among women in rural pastoralist community of hamar district, southern Ethiopia: a case–control study. Risk Management and Healthcare Policy 2020:2159-2167.

3.	Zegeye EA, Reshad A, Bekele EA, Aurgessa B, Gella Z: The state of health technology assessment in the Ethiopian health sector: learning from recent policy initiatives. Value in Health Regional Issues 2018, 16:61-65.

**Thank you**
