# Supplementary material for: Antenatal care booked rural residence women have home delivery during the era of COVID-19 pandemic in Gidan District, Ethiopia
Source: PLoS One. 2023 Dec 5;18(12):e0295220. doi: 10.1371/journal.pone.0295220 (PMC10697573; doi:10.1371/journal.pone.0295220)
Supplement: Attachment — Submitted filename: Response to Reviewers.docx [file pone.0295220.s008.docx]

Authors’ response to the reviewer’s comments

Title**:** Antenatal care booked rural residence women have high home delivery during the era of COVID-19 Pandemic in Gidan District, Ethiopia

Authors

Desale Bihonegn Asmamaw ([desalebihonegn1988@gmail.com](mailto:desalebihonegn1988@gmail.com))

Yohannes Ayanaw Habitu (([yohaneshabitu@gmail.com](mailto:yohaneshabitu@gmail.com))

Eskedar Getie Mekonnen ([eskedargetie18@gmail.com](mailto:eskedargetie18@gmail.com))

Wubshet Debebe Negash ([wubshetdn@gmail.com](mailto:wubshetdn@gmail.com))

Date: 22/04/022023

Authors’ Point-by-point response

Dear reviewer, we would like to extend our deepest appreciation for devoting your time to reviewing our manuscript entitled "Antenatal care booked rural residence women have high home delivery during the era of COVID-19 pandemic in Gidan District, Ethiopia". The whole structure of the manuscript has been revised. We hope the manuscript is now clearer and more acceptable than its previous version. We have tried to present the paper in the proper manner according to your comment on how to do so. For this reason, we have given our responses to each of the concerns you raised.

Reviewer comments

Reviewer comments: Which have high percentage E.g., unmarried women home delivery, rural residents home delivery, husband education with home delivery and not involving in women development army and home delivery or fear of COVID 19 and home delivery i.e. Why you prefer unmarried women over others? Taking the culture of the community and the occurrence of conflict in the area how do you say that most of them were unmarried since it is uncommon for the women to be pregnant to these much number? If that is so is that wanted pregnancy?

Authors’ response: Dear reviewer, thank you for your concerns and suggestions. We appreciate your point of view; yes, you are right, and we modified the topic accordingly. We prefer rural residence women based on consideration of the odds ratio, the culture of the community, the occurrence of conflict in the area, and the accessibility of the services. Kindly see the updated version of the manuscript.

Reviewer comments: I prefer the title to be “ANC Booked Unmarried women have high Home delivery during the era of COVID 19 Pandemic in Gidan District, Ethiopia

Authors response: Dear reviewer, thank you for your suggestions. The title was reframed as accordingly. Kindly see the updated version of the manuscript.

Reviewer comments: Background: It is well refined but still needs strengthening and shortening to the main objective of the study

Authors’ response: Dear reviewer, thank you for your observations and comments. This has been addressed in detail. Kindly see the updated version of the manuscript.

Reviewer comments: The study period was not well written i.e., “March 30 and April 29, 2021”

Authors response: Thank you for your suggestion and observations. This has been corrected, kindly see the update version of it.

Reviewer comments: Have you conducted training of the data collectors to maintain quality assurance?

Authors’ response: Yes, the data collectors and supervisors were trained for two days, focusing on how to ask and fill out the questionnaires, the selection criteria for women, and how to approach the respondents. Kindly see page 9, line 144-147.

Reviewer comments: Are there supervisors for the data collectors? How one PI supervisors over 760 sample?

Authors response: There are eight BSc midwifery or nursing data collectors and two supervisors in the same field with experience in research and fieldwork coordination who participated in the data collection process. The whole data collection process was closely supervised by the principal investigator (PI). Kindly see page 9, line 160-163.

Reviewer comments: Who translated the two forms of the questionnaire?

Authors response: Thank you for your concerns. The two forms of the questionnaires were translated by language experts who have previous experience with this.

Reviewer comments: In the analysis have you calculated the case to the variable ratio? If so, what was the result?

Authors’ response: Yes, we calculated the case-to-variable ratio, the case-to-variable ratio showed 12.8 to 1, which indicates a ratio above the required 5 to 1. Kindly see the updated version of the manuscript.

Reviewer comments: The tables are not self-explanatory E.g. Didn’t describe the district.

Authors response: Thank you for your comments, this has been addressed, kindly see the updated tables.

Reviewer comments: The discussion needs more refinement, There should be theoretical, practical, logical and frame work related explanations for those findings.

Authors response: Dear reviewer, thank you for suggestions and comments, this has been addressed; kindly see the updated version of the manuscript.

Reviewer comments: Would you cross tab those associated factors with the home delivery.

Authors response: Dear reviewer, thank you for your concerns, this has been already done, kindly see table 3.

Reviewer comments: The conclusion should be drawn from the objective and implies the main issue of the study.

Authors response: Dear reviewer, thank you for your comments and suggestions, this has been addressed.

Reviewer comments: Where is whether there is funding or not.

Authors response: This is available in the declaration section, kindly see page 17, line 313-314.

Reviewer comments: Use of words: E.g., ‘North east Ethiopia’ should be Northeastern Ethiopia

Capital Letters E.g., kilometers should be Kilometers

Slided message: E.g., "who were booked for ANC"

Some of the figures lack percentage after them E.g., "from 16 to 33%."

Inconsistencies E.g., Use of institutional delivery and health facility delivery and giving birth at health facilities and health institution delivery.

Sentence flow gaps E.g., COVID-19 have disastrous effect.

Authors response: Thank you for your observations and suggestions, these have been corrected and addressed, kindly see the updated version of the manuscript.

Reviewer comments: Still careful review on the fitness of the model, analysis and interpretations is needed.

Authors response: Thank you for your comments, we were done the Hosmer-Lemeshow test, the model was adequate with the p-value of 0.26. Kindly see the updated version of the manuscript.

Reviewer comments: Language and Grammar

Authors response: Thank you for the suggestion, the entire article has been read and corrected by a native English speaker. Kindly see the updated version of the manuscript.

**Thank you**
