# Supplementary material for: Antenatal care booked rural residence women have home delivery during the era of COVID-19 pandemic in Gidan District, Ethiopia
Source: PLoS One. 2023 Dec 5;18(12):e0295220. doi: 10.1371/journal.pone.0295220 (PMC10697573; doi:10.1371/journal.pone.0295220)
Supplement: Attachment — Submitted filename: Response to Reviewers.docx [file pone.0295220.s009.docx]

Authors’ response to the reviewer’s comments

Title**:** Antenatal care booked rural residence women have home delivery during the era of COVID-19 Pandemic in Gidan District, Ethiopia

Authors

Desale Bihonegn Asmamaw ([desalebihonegn1988@gmail.com](mailto:desalebihonegn1988@gmail.com))

Yohannes Ayanaw Habitu (([yohaneshabitu@gmail.com](mailto:yohaneshabitu@gmail.com))

Eskedar Getie Mekonnen ([eskedargetie18@gmail.com](mailto:eskedargetie18@gmail.com))

Wubshet Debebe Negash ([wubshetdn@gmail.com](mailto:wubshetdn@gmail.com))

Date: 22/04/022023

Authors’ Point-by-point response

Dear reviewer, we would like to extend our deepest appreciation for devoting your time to reviewing our manuscript entitled "Antenatal care booked rural residence women have home delivery during the era of COVID-19 pandemic in Gidan District, Ethiopia". The whole structure of the manuscript has been revised. We hope the manuscript is now clearer and more acceptable than its previous version. We have tried to present the paper in the proper manner according to your comment on how to do so. For this reason, we have given our responses to each of the concerns you raised.

Reviewer comments

Reviewer comments:  , ...Despite the importance of giving birth at a health institution, in Ethiopia, according to the Ethiopian Demographic Health Survey report, nearly half of the ANC-booked mothers gave birth at home do not you think that the finding of EDHS data report of home birth is higher than the finding of this study? therefore, please omit the term high from the title of this research , for even it is below the national report, EDHS!

Authors response: Dear reviewer, thank you for your concerns, we have been addressed this issue according to your comments, kindly see the updated version of the manuscript.

Reviewer comments: I don think that the sampling procedure is multi stage sampling because no more than one stage was gone to gate sampling unit for this study, u started from woreda then no more step to reach sampling unit simple u can select study unit SRS being at woreda level or kebeles due to the list already known and sharing the sample size proportionally for srata u randomly selected. there fore modify to simple random sampling.

Authors response: Dear reviewer, thank you for your observation and suggestion, we have been modified according to your comment and suggestion, kindly see the updated version of the manuscript.

Reviewer comments: repeated statements are there eg model adequacy

Authors’ response: Dear reviewer, thank you for your observations, the errors were corrected, kindly see the updated versions of the manuscript.

Thank you
